# Vacuum Plates

**Published:** 1887-11

**Authors:** W. E. Swigert

**Affiliations:** Spencer, Ind.


ARTICLE III.

### "VACUUM" PLATES.

BY W. E. SWIGERT, D. D. S., SPENCER, IND.

A lady once told me that the reason her mother couldn't control her upper plate was, because I hadn't made the "air chamber" in the right place, and it was the wrong shape. She had examined several mouths and found that Nature had provided a portion of the palate especially for an "air chamber," had made it a peculiar shape, and I had failed to fit it. The blow staggered me. I was dumb with amazement, and found great beads of sweat "slowly and sadly" trickling down my *manly* face, and a ghastly smile o'ershadowed my ghost-like countenance. I could but realize and admire the great truth that a superior mind, in one short observation, had discovered what I had failed to in all the years I had practiced dentistry, and I exclaimed, "Success to thy genuine, Oh, Woman!"

But, seriously, is the vacuum really a good thing? Rather, is it not a myth and delusion? Aye, more, in some cases it is an absolute injury. I cannot account for the persistent use of the vacuum plate by so many dentists, on any other ground than that of ignorance, or, rather want of disposition to investigate.

The per cent. of dentists who follow in the old rut of their preceptors, who make dentistry a mere trade, to be worked at only for the money, who care not for the best methods, but are willing to accept the first given as good enough without further investigation, is surprisingly large.

Take, for example, the air chamber. How many of our "shining lights" will persist in using it in all artificial dentures? And what per cent. of them can give an intelligent reason for so doing?

I believe the principle upon which an exhaustion plate is made *will condemn the vacuum.* What is necessary to secure the most perfect adhesion to the mouth? I reply, *perfect adaptation.* By this I do not mean to merely take a good impression, with the parts in their natural position, and make the plate fit the model taken from this. Far from it. In the majority of cases such a plate will not fit, because there is not perfect adaptation. Perfect adaptation is when a plate is in place in the mouth and the air exhausted there will be an equal pressure over the entire surface, thereby producing a perfect adhesion. Hence, in the majority of mouths, there will not be a perfect fit, because the pressure will not be distributed equally, for the reason that in all mouths will be found inequality of texture, and the plate will press harder on the more solid tissues than on the softer. By examining a few mouths any one can easily satisfy himself of this fact. Examine the palate of a mouth, posterior to the plate, and what is found true there will be found true of other parts where there is an inequality of tissues. I have seen plates, because of the unequal pressure, that would almost bruise the harder parts, while they barely touched the softer. Not because of a bad model, but for the reason given above.

How to equalize this pressure and secure perfect adaptation is the important question here. I would suggest that we study well the mouth, going over it again and again, using pressure to find what parts are soft. Note in what direction the soft tissues should be pressed, to be left in as easy and natural a position as is possible to have an equalized pressure and adhesion. Determine the extent to which they should be forced. Keep in mind the fact that too much pressure on the soft parts will cause correspondingly less on the harder, thereby making as imperfect a fit as the reverse. Take the model, which should be a perfect counterpart of the mouth, with all tissues in a perfectly natural position, and note on it all the inequalities. Study the model and mouth together, until as familiar with the model as though looking at the mouth. For an accurate fit will now depend upon the work done on the model. With a suitable scraper pare off the model where the tissues are soft in the mouth until satisfied that adhesion will be as strong as on the harder parts, remembering the direction in which the tissues in the mouth should be pressed, and retaining as near as possible the natural shape of the model.

To obtain a perfect result, will not only require good judgment, but a good stock of patience, perseverance and practice. Every failure will be only the forerunner of a victory. Do not expect to become perfect in a few trials, or disappointment will be the result; for, to obtain a perfectly equal pressure and adhesion, I believe is only a thing of theory and probably impossible to gain, but strive to reach it and the result will be a surprise.

If perfect adaptation is necessary, I do not think it is obtained with a vacuum. An equal pressure and adhesion over the entire surface of the plate can be obtained only by equalizing the firmness or solidity of the tissues. Is this done with a vacuum? No. A plate with a vacuum will pull the soft parts down to the chamber, drawing them from under the plate, where it is made to fit, thereby changing, to a certain extent, the shape of the mouth from the shape of the plate. And how often do we see, in cases of this kind, a distorted, inflamed, and honeycombed or papulous condition of this portion of the mouth?

[This subject is not new; but it is not hackneyed, and is in order till common sense shall govern us in the adaptation of plates.—ED. JOURNAL.]—*The Ohio Journal of Dental Science.*

